# Improving cohort coverage estimation using a data triangulation framework: the Swiss HIV Cohort Study example

**DOI:** 10.1097/QAD.0000000000004523

**Published:** 2026-04-10

**Authors:** Jessy J. Duran Ramirez, Roger D. Kouyos, Irene Abela, Charles Beguelin, Marco Bongiovanni, Philip Bruggmann, Alexandra Calmy, Matthias Cavassini, Alexia Cusini, Mirjam de Roche, Luigia Elzi, Andrée Friedl, Rosamaria Fulchini, Chistoph Fux, David Haerry, Benjamin Hampel, Markus Herold, Philipp Kaiser, Helen Kovari, Patrick Schmid, Delphine Sculier, Marcel Stöckle, Philip E. Tarr, Frédéric Tissot, Jonathan Tschopp, Gilles Wandeler, Andri Rauch, Huldrych F. Günthard, Katharina Kusejko

**Affiliations:** a Department of Infectious Diseases and Hospital Epidemiology, University Hospital Zurich; b Institute of Medical Virology, University of Zurich, Zurich; c Department of Internal Medicine, Hospital Center of Bienne, Bienne; d Department of Infectious Diseases, Inselspital, Bern University Hospital, University of Bern, Bern; e Division of Infectious Diseases, Ente Ospedaliero Cantonale, Lugano; f Arud Centre for Addiction Medicine, Zurich; g HIV Unit, Department of Infectious Diseases, Geneva University Hospitals, Geneva; h Department of Infectious Diseases, Lausanne University Hospital; i University of Lausanne, Lausanne; j Division of Infectious Diseases, Cantonal Hospital of Grisons, Chur; k Infectious Diseases, Department of Internal Medicine, Spital STS AG, Thun; l Division of Infectious Diseases, Regional Hospital Bellinzona, Bellinzona; m University of Basel, Basel; n Division of Infectious Diseases and Infection Prevention, Cantonal Hospital Baden, Baden; o Kantonsspital Münsterlingen, Münsterlingen; p Department of Infectious Diseases and Infection Prevention, Kantonsspital Aarau; q Chair Positive Council; r Department of Public and Global Health, Epidemiology, Biostatistics and Prevention Institute, University of Zurich; s Checkpoint Zurich, Zurich; t Maihofpraxis, Lucerne; u Division of Infectious Diseases and Infection Prevention, Cantonal Hospital Luzern, Luzern; v Center for Infectious Diseases, Klinik im Park, Zurich; w Division of Infectious Diseases, Infection Prevention and Travel Medicine, HOCH Health Ostschweiz, Cantonal Hospital St. Gallen, University Teaching and Research Hospital, St. Gallen; x Private Practice Office, Geneva; y Division of Infectious Diseases and Hospital Epidemiology, University Hospital Basel, University of Basel, Basel; z University Center for Internal Medicine, Infectious Diseases and Hospital Epidemiology Service, Kantonsspital Baselland, University of Basel, Bruderholz; aa Private Practice, Lausanne, Switzerland.

**Keywords:** cohort coverage, cohort study, data triangulation, HIV epidemic, HIV surveillance, representativeness

## Abstract

**Objectives:**

To improve estimation of cohort coverage in the Swiss HIV Cohort Study (SHCS) using a data triangulation framework that compares SHCS data with multiple external data sources.

**Design:**

Retrospective longitudinal analysis of the SHCS.

**Methods:**

Cohort coverage of HIV diagnoses, AIDS diagnoses, and antiretroviral therapy (ART) uptake was triangulated across SHCS data (1985–2023), national HIV/AIDS surveillance (1985–2023), longitudinal antiretroviral therapy (ART) sales data (2017–2024), and a targeted literature comparison. Temporal trends in cohort coverage were assessed, and demographic representativeness was evaluated by sex, age, HIV acquisition mode, and region. Mean cohort coverage estimates were calculated for three outcomes: HIV and AIDS diagnoses (Step 1) and ART uptake (Step 2).

**Results:**

Over 38 years, mean SHCS coverage was 62.4% for HIV diagnoses, 74.0% for AIDS, and 64.9% for ART uptake, consistent with literature-based estimates. Coverage of HIV diagnoses declined in recent years, and geographical heterogeneity was observed. The SHCS remained broadly representative across most subgroups; however, females, older adults, and people with heterosexually acquired HIV or using psychoactive substances were underrepresented, while people on single-tablet and salvage ART regimens were overrepresented.

**Conclusions:**

A data triangulation framework provides a practical approach for monitoring cohort coverage and representativeness. While the SHCS captures a broadly representative sample of diagnosed individuals, tailored strategies are needed to improve inclusion of underrepresented subgroups and maintain cohort coverage. Sustained monitoring is essential to ensure that cohort-based research remains generalizable and continues to inform clinical care and public health responses in Switzerland and beyond.

## Introduction

Accurate estimation of cohort coverage is essential to ensure that findings from national cohort studies are representative and generalizable. In epidemiology, cohort coverage refers to the proportion of individuals included in a study relative to the reference population [1]. Complete cohort coverage is rarely achievable because of logistical challenges, regulatory restrictions, and uncertainty about the true population size. High cohort coverage does not necessarily ensure representativeness, which requires that included individuals reflect the characteristics of the reference population [2].

Estimating cohort coverage is particularly challenging in the context of the human immunodeficiency virus (HIV) epidemic in Switzerland, as in many other countries [3]. First, the total number of people with HIV (PWH), including those undiagnosed, is uncertain and relies on model-based estimates [4]. Second, privacy restrictions and data anonymization prevent individual-level linkage across data sources. This limits the assessment of duplication of reported diagnoses, migration dynamics (e.g., whether people diagnosed in Switzerland remain in Switzerland), and disengagement from care. Third, certain groups – including women, migrants, ethnic minorities, and socially marginalized populations – may also be underrepresented due to differences in healthcare access, health seeking behaviour, administrative barriers, and structural factors such as stigma, and discrimination [5–8]. These factors can bias estimates, reduce generalizability, and affect the allocation of healthcare resources [9–12]. Finally, cohort coverage may also vary over time or across subgroups depending on data sources and denominator definitions [9,13–16].

Data triangulation provides a practical approach to address these challenges. By comparing multiple independent data sources, data triangulation allows cross-validation, mitigates biases inherent in individual sources, and clarifies discrepancies across estimates [1,17].

The Swiss HIV Cohort Study (SHCS, www.shcs.ch) provides an ideal setting for applying this approach, given its well-defined population of people diagnosed with HIV in Switzerland and its comprehensive longitudinal data collection [13,15,18]. In this study, we define data triangulation as the comparison of SHCS data with independent external data sources. HIV and AIDS are notifiable diseases in Switzerland, requiring reporting from all Swiss laboratories and physicians to the Federal Office of Public Health (FOPH), and the national surveillance therefore provides a comprehensive record of diagnosed individuals. In addition, national antiretroviral therapy (ART) sales data provide an independent measure of ART uptake at the population level. This study aimed to improve cohort coverage estimation and to assess cohort coverage and representativeness using a data triangulation framework.

## Methods

### Data sources

The SHCS is a nationwide, multicentric cohort that prospectively enrols adult PWH in Switzerland using a standardized protocol since 1988 [15]. Participating centres had collected clinical information using a similar protocol since HIV screening tests became available in 1985; these data were incorporated into the SHCS. A small number of well-documented PWH were retrospectively included, with records dating back to 1982. Data from years before 1985 were grouped into a single category (≤1985) because of small numbers and to align with the NSR. By June 2025, 22 282 participants had been enrolled from university and cantonal hospitals, and, since 1996, also from regional hospitals and private practitioners (Figure S1). Participation is voluntary for both healthcare providers and PWH.

The SHCS and all associated study protocols have been approved by the ethics committee (BASEC-Number 2023-02080), and participants have provided written informed consent. Sociodemographic and clinical data are collected longitudinally and pseudonymized using a unique identifier [15,16]. The Swiss Mother and Child HIV Cohort Study (MoChiV) collects perinatal HIV data and transitions participants to the SHCS at adulthood [19].

National HIV and AIDS surveillance data were obtained from the FOPH. HIV and AIDS are notifiable diseases in Switzerland, and laboratories and physicians report each diagnoses along with minimal demographic information for patient de-duplication (Supplementary Material). The resulting “national surveillance report” (NSR) is aggregated annually for 1985–2023.

Anonymous ART sales data were provided by Gilead Sciences with consent from the supplying pharmaceutical companies to IQVIA AG (Switzerland, www.iqvia.com), which collects data from pharmacies and wholesalers. These data cover monthly ART sales from June 2017 to May 2024.

## Data triangulation framework

We applied a three-step data triangulation framework:

### Step 1: National surveillance report to estimate SHCS coverage for people diagnosed with HIV or AIDS

HIV diagnosis was defined as the earliest available evidence of infection in the SHCS, including a documented or self-reported positive test, SHCS enrolment, or the occurrence of an AIDS-defining condition (Category C event, according to the U.S. Centers of Disease Control and Prevention classification [20]). AIDS diagnosis was defined as the first occurrence of an AIDS-defining condition.

Cohort coverage was estimated using SHCS-to-NSR ratios. Median HIV/AIDS coverage was calculated as median of annual ratios, with corresponding interquartile ranges (IQR). Total HIV/AIDS coverage was calculated as total SHCS participants with HIV/AIDS-defining conditions relative to NSR totals.

### Demographical stratification

Analyses were stratified according to NSR categories, with SHCS data harmonized accordingly. Sex assigned at birth was categorized as male or female; individuals with missing or unknown sex were excluded (Figure S2, Supplemental Digital Content). Treatment eras were defined to reflect changes in ART guidelines: 1985–1986 (pre-treatment era; no ART), 1987–1994 (pre-HAART era; ART available and start of SHCS enrolment), 1995–2007 (HAART era; introduction of three-drug combination therapy), 2008–2018 (“treat-all” era; gradual adoption of ART regardless of CD4^+^ cell count [21]), 2019–2023 (recent ART era; dual regimens emphasized). Recent data on gender identity (since 2020), including non-binary categories (since 2023), were insufficient to allow separate analysis of transgender participants [22].

Canton of residence were grouped into seven major regions according to Swiss Federal Statistical Office's classification: Lake Geneva Region, Midland, Northwestern Switzerland, Zurich, Eastern Switzerland, Central Switzerland, and Ticino (Figure S3, Supplemental Digital Content) [23]. The region at enrolment was used as a proxy for the region at HIV/AIDS diagnosis, as changes during follow-up were rare (2.5%). Total regional coverage was calculated for 2014–2023 and separately by year. Age at HIV/AIDS diagnosis was calculated from year of birth and diagnosis, then grouped in 10-year intervals (0–9, …, ≥70 years). Participants diagnosed before age 20 (824 participants; 3.7% of the SHCS) and those with missing or unknown age were excluded to align with SHCS eligibility and NSR (Figure S4, Supplemental Digital Content).

Modes of HIV acquisition were categorized based on patient-reported sources: men who have sex with men (MSM; including gay, bisexual, and other men who have sex with men), heterosexual contact (HET), people who inject psychoactive substance (PWIPS), and individuals infected via haemophilia, transfusions, or perinatal transmission. Participants with missing, unknown, or other acquisition modes were excluded (Figure S5, Supplemental Digital Content). Demographical representativeness was assessed using SHCS-to-NSR proportional ratios for 1985–2023 and separately by year. Ratios >1 indicated over-representation and ratios <1 indicated under-representation.

### Sensitivity analyses

Sensitivity analyses were performed by assessing the impact of annual fluctuations in reporting or diagnoses using cumulative median HIV/AIDS coverage; evaluating the influence of participant-reported binary gender; examining the impact of delayed SHCS enrolment through comparison with a 2021 SHCS dataset; assessing potential misreporting of first AIDS-defining conditions by examining multiple events and inter-event intervals; and evaluating demographic representativeness using SHCS-to-NSR proportional ratios for 2000–2023 and 2010–2023, accounting for changes in FOPH reporting.

### Step 2: Antiretroviral therapy sales to estimate SHCS coverage for ART

The analysis focused on the five most often prescribed single-tablet regimens in the SHCS as of 31 December 2023: Biktarvy (bictegravir/emtricitabine/tenofovir alafenamide; BIC/FTC/TAF; 28.4%), Dovato (dolutegravir/lamivudine; DTG/3TC; 19.1%), Odefsey (rilpivirine/emtricitabine/tenofovir alafenamide; RPV/FTC/TAF; 8.4%), Triumeq (abacavir/dolutegravir/lamivudine; ABC/DTG/3TC; 5.6%), and Genvoya (elvitegravir/cobicistat/emtricitabine/tenofovir alafenamide; EVG/COBI/FTC/TAF; 4.9%). Together, these five regimens accounted for 66.4% of all ART prescriptions in the SHCS (Table S1, Supplemental Digital Content, www.shcs.ch/about-shcs/shcs-key-data-tables/). In addition, we analysed compounds often used in “salvage regimens”, which are prescribed when standard treatments are no longer effective or well tolerated. These included Prezista (darunavir, DRV; 4.7%), Intelence (etravirine, ETV; 0.5%), Celsentri (maraviroc, MVC; 0.2%) and Retrovir (zidovudine, ZDV; 0.1%). Their use was interpreted as a proxy for the burden of treatment-experienced or heavily pre-treated individuals.

### Workflow and analysis

Swiss ART sales data are reported by package, whereas SHCS prescription data are recorded at the individual-level. A structured workflow was therefore implemented to convert prescriptions into monthly package estimates and compared them with Swiss ART sales data (Supplementary Material). Total ART coverage from June 2017 to May 2024 and median monthly ART coverage per regimen were then calculated.

### Step 3: Targeted literature comparison

All SHCS-published studies reporting cohort coverage estimates for reference populations in Switzerland were included, regardless of methodology or comparison group, provided they used a national denominator. Key study characteristics were extracted and summarized, including analysis period, reported cohort coverage estimates, and methodological context.

Mean cohort coverage estimates were calculated for three outcomes: people diagnosed with HIV and people diagnosed with AIDS (Step 1), and people on ART (Step 2). To enable direct comparison with published estimates, analyses were restricted to the time frames reported in each study. All analyses were conducted using R, version 4.5.2.

## Results

### Comparison with national surveillance reports

**Fig. 1:**
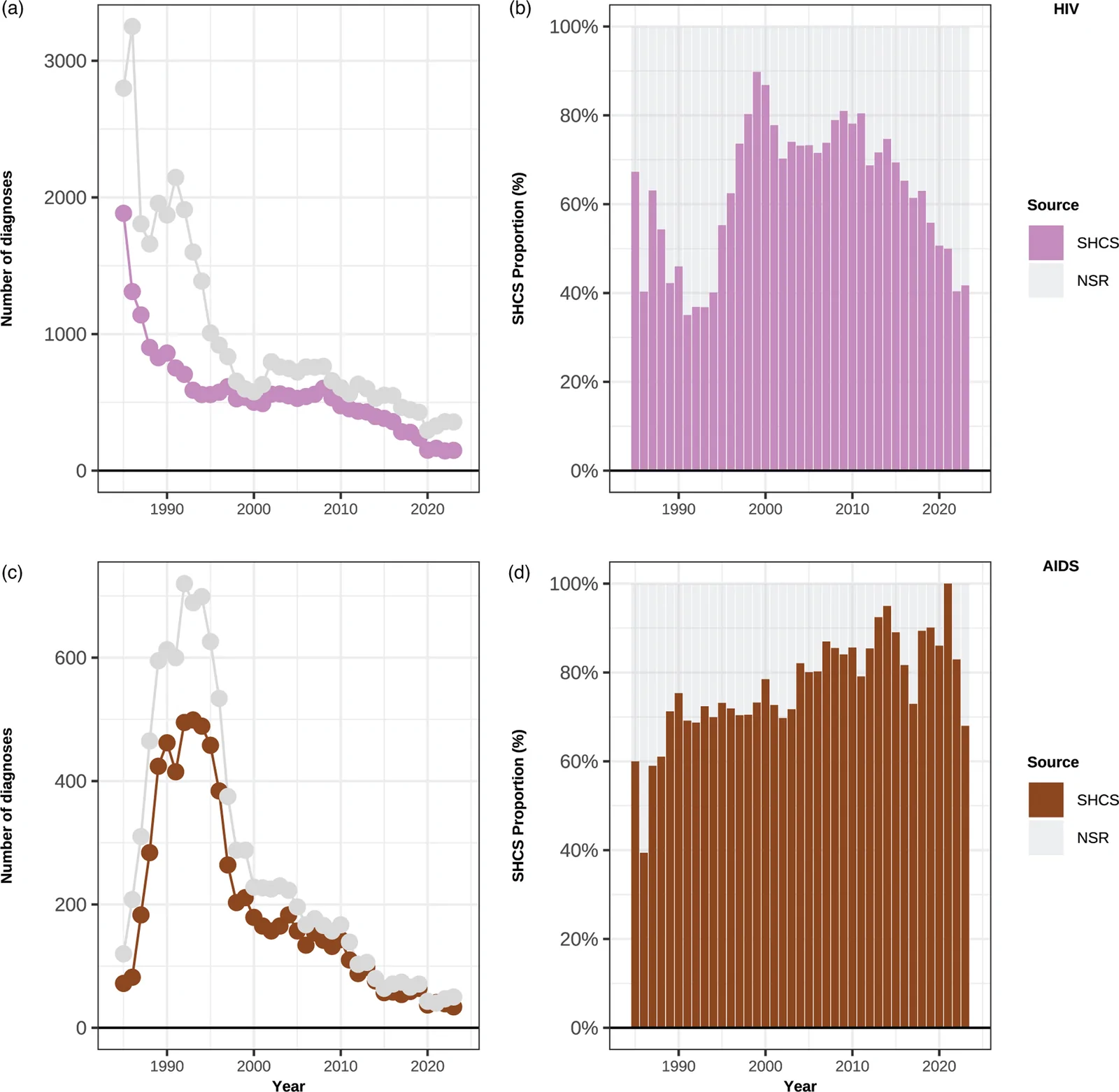
Annual numbers (a, c) and proportions (b, d) of people diagnosed with HIV and of people diagnosed with AIDS-defining conditions, 1985–2023.

As of December 31, 2023, the SHCS included 22 110 PWH, of whom 7451 had ever been diagnosed with an AIDS-defining condition (Figure S1, Supplemental Digital Content). Annual HIV diagnoses in the NSR were generally higher than in the SHCS (Fig. 1a).

SHCS data (purple for HIV, brown for AIDS) are compared to NSR data (grey). Panels a and c show number of diagnoses. Panels b and d show the annual SHCS-to-NSR ratios, representing annual SHCS coverage. The *x*-axis shows year of diagnosis or report. Abbreviations: SHCS, Swiss HIV Cohort Study; NSR, National Surveillance Report.

SHCS coverage of HIV diagnoses ranged from 67% in 1985, peaked at 90% in 1999, and declined to 42% in 2023 (Fig. 1b). Median annual HIV coverage was 67% [interquartile range (IQR) 50.3–73.9%], and total coverage was 57.7% (22 110 SHCS/38 294 NSR).

SHCS coverage of AIDS-defining conditions was generally higher and mostly increasing over time (Fig. 1c, d), with median annual AIDS coverage of 75.4% (IQR 70.4–85.5%) and total AIDS coverage of 72.7% (7451/10 247).

### Demographic stratification and representativeness

**Fig. 2:**
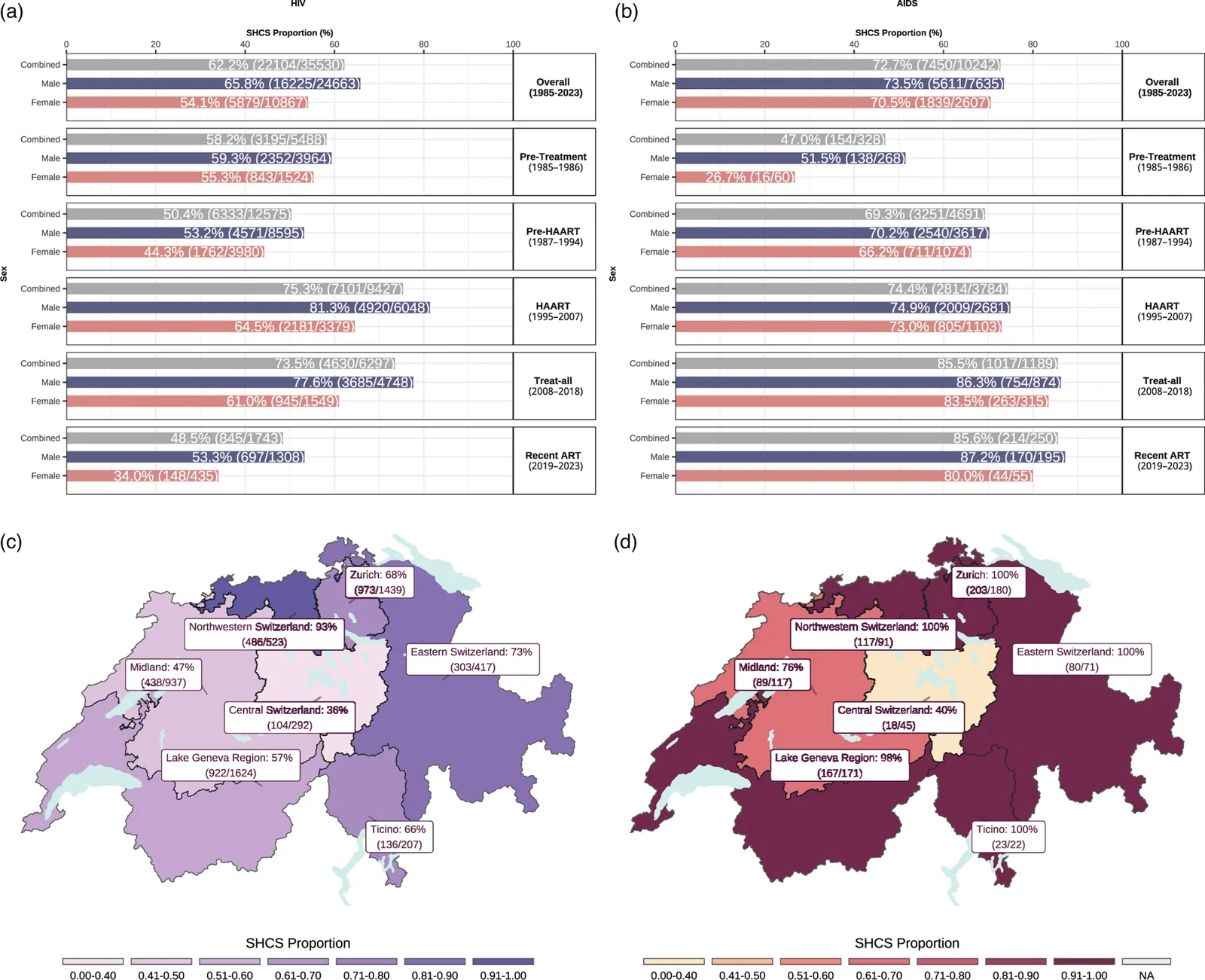
Time trends in combined, sex-specific (a, b), and regional (c, d) HIV and AIDS diagnoses coverage.

Median annual HIV coverage was higher among males (73.1%, IQR 57.9–82.6%) than females (57.7%, IQR 44.0–65.3%; Figure S6A, Supplemental Digital Content). Total HIV coverage was also higher among males than females (65.8% vs. 54.1%), with the largest difference in the recent ART era (53.3% vs. 34.0; Fig. 2a). Males were overrepresented among HIV diagnoses in the SHCS compared with the NSR (73.4% SHCS/64.4% NSR, ratio 1.14, *n* = 16 225 SHCS and 24 663 NSR) and females underrepresented (26.6% SHCS/28.4% NSR, ratio 0.94, *n* = 5879 SHCS and 10 867 NSR; Table S2, Figure S7A, B, Supplemental Digital Content).

(a, b) Sex-specific coverage of HIV (left) and AIDS (right) diagnoses (in percentage) is shown for combined (grey), males (blue), and females (red), grouped into overall and five treatment eras from 1985 to 2023. The *x*-axis represents SHCS coverage; *y*-axis lists the treatment eras; numbers within the bars indicate SHCS coverage in percentage, with the total number of SHCS diagnoses divided by total NSR diagnoses shown in parentheses. (c, d) Total regional SHCS coverage of HIV (purple) and AIDS (brown) diagnosis from 2014 to 2023. Major region of residence coloured by total regional SHCS coverage. The number after each major region name indicates the total regional SHCS coverage in percentage, with the corresponding total SHCS divided by total NSR in parentheses. Due to differences in diagnosis year and reporting year, total SHCS counts may exceed total NSR counts, resulting in cohort coverage values >100%. Abbreviations: SHCS, Swiss HIV Cohort Study; NSR, National Surveillance Report.

Similarly, median annual AIDS coverage was higher among males (78.4%, IQR 70.5–84.3%) than females (71.3%, IQR 67.6–84.2%; Figure S6B, Supplemental Digital Content). Total AIDS coverage was also higher among males (73.5% vs. 70.5%), particularly in the pre-treatment era (51.5% vs. 26.7%; Fig. 2b). Males were also slightly overrepresented among AIDS diagnoses (75.3% SHCS/74.5%, ratio 1.01, *n* = 5611 SHCS and 7635 NSR) and females underrepresented (24.7% SHCS/25.4% NSR, ratio 0.97, *n* = 1839 SHCS and 2607 NSR; Table S2, Figure S7C, D, Supplemental Digital Content).

Total HIV or AIDS diagnoses coverage varied considerably across major regions, both as aggregated 10-year period and by year (Fig. 2c, d, Figure S8A, B, Supplemental Digital Content). The highest total HIV regional coverage was observed in Northwestern Switzerland (93%), followed by Eastern Switzerland (73%) (Fig. 2c). For AIDS diagnoses, five major regions had SHCS coverage levels exceeding 98% (Fig. 2d).

SHCS coverage varied by age and, to a lesser extent, by HIV acquisition mode (Figures S9A, B and S10A, B, Supplemental Digital Content). Median annual HIV coverage was highest among individuals aged 20–29 (100%, IQR 94.7–100%) and declined to 58.6% (IQR 37.5–75.0%) among individuals aged ≥70, with a similar but lower pattern for AIDS coverage (Figure S9A, B, Supplemental Digital Content). Individuals aged 20–29 and those with MSM acquisition were overrepresented in the SHCS compared with the NSR, whereas older individuals (≥40 years) and those with HET or PWIPS acquisition were underrepresented (Table S2, Figure S11, Supplemental Digital Content).

### Sensitivity analyses

Sensitivity analyses based on cumulative annual median coverage (Figure S12, Supplemental Digital Content) and current gender led to similar results (data not shown). Comparison with the 2021 SHCS dataset confirmed the observed decline in recent HIV coverage, suggesting that this trend was not driven by delayed enrolment (Figure S13, Supplemental Digital Content). Examination of multiple AIDS-defining conditions in individuals (4203/7451, 56.1%) showed that most events (91.4%) occurred on different dates within one year (median 86 days, IQR 2–327), supporting the robustness of AIDS classification (Figure S14, Supplemental Digital Content). Patterns of demographic representativeness were consistent, with overrepresentation of males, individuals aged 20–29 and those with MSM or perinatal HIV acquisition, and underrepresentation of females, individuals aged ≥40, those with HET or PWIPS acquisition (Table 1, Table S3, Supplemental Digital Content).

**Table 1:** Demographical representativeness for 2000–2023 and 2010–2023

Variable		*N* SHCS	*N* NSR	SHCS %	NSR %	Ratio SHCS%/ NSR %^a^
**HIV 2000–2023**						
Sex at birth	Female	2382	4034	24.37	29.05	0.84
	Male	7386	9700	75.58	69.85	1.08
Acquisition mode	MSM	4977	4355	53.61	41.07	1.31
	HET	3807	5335	41.01	50.32	0.82
	PWIPS	330	749	3.55	7.06	0.50
	Perinatal	108	87	1.16	0.82	1.42
	Haemophilia	0	0	0.00	0.00	
	Transfusion	61	77	0.66	0.73	0.90
Age groups (years)	20–29	2670	2594	28.11	22.73	1.24
	30–39	3443	4086	36.25	35.81	1.01
	40–49	2020	2670	21.27	23.40	0.91
	50–59	945	1388	9.95	12.16	0.82
	60–69	358	548	3.77	4.80	0.78
	70+	61	125	0.64	1.10	0.59
**AIDS 2000–2023**						
Sex at birth	Female	712	894	28.20	28.73	0.98
	Male	1813	2218	71.80	71.27	1.01
Acquisition mode	MSM	969	918	40.58	31.89	1.27
	HET	1106	1456	46.31	50.57	0.92
	PWIPS	266	465	11.14	16.15	0.69
	Perinatal	19	15	0.80	0.52	1.53
	Haemophilia	4	0	0.17	0.00	
	Transfusion	24	25	1.01	0.87	1.16
Age groups (years)	20–29	279	308	11.18	9.97	1.12
	30–39	803	951	32.17	30.80	1.04
	40–49	749	966	30.01	31.28	0.96
	50–59	414	537	16.59	17.39	0.95
	60–69	193	241	7.73	7.80	0.99
	70+	58	85	2.32	2.75	0.84
**HIV 2010–2023**						
Sex at birth	Female	820	1603	18.88	23.88	0.79
	Male	3519	5031	81.01	74.93	1.08
Acquisition mode	MSM	2514	2440	61.71	50.10	1.23
	HET	1433	2213	35.17	45.44	0.77
	PWIPS	58	151	1.42	3.10	0.46
	Perinatal	46	41	1.13	0.84	1.34
	Haemophilia	0	0	0.00	0.00	
	Transfusion	23	25	0.56	0.51	1.10
Age groups (years)	20–29	1143	1126	26.93	20.22	1.33
	30–39	1373	1784	32.35	32.04	1.01
	40–49	941	1365	22.17	24.52	0.90
	50–59	559	898	13.17	16.13	0.82
	60–69	193	316	4.55	5.68	0.80
	70+	35	79	0.82	1.42	0.58
**AIDS 2010–2023**						
Sex at birth	Female	223	279	23.28	24.89	0.94
	Male	734	838	76.62	74.75	1.02
Acquisition mode	MSM	423	385	47.10	39.53	1.19
	HET	403	513	44.88	52.67	0.85
	PWIPS	59	66	6.57	6.78	0.97
	Perinatal	7	5	0.78	0.51	1.52
	Haemophilia	2	0	0.22	0.00	
	Transfusion	4	5	0.45	0.51	0.87
Age groups (years)	20–29	89	95	9.37	8.53	1.10
	30–39	220	241	23.16	21.63	1.07
	40–49	279	339	29.37	30.43	0.97
	50–59	230	281	24.21	25.22	0.96
	60–69	99	117	10.42	10.50	0.99
	70+	33	41	3.47	3.68	0.94

^a^ >1 overrepresentation, <1 underrepresentation, ~1 similar representation.

HET, heterosexual contact; MSM, men who have sex with men; NSR, National Surveillance Report; PWIPS, people who inject psychoactive substance; SHCS, Swiss HIV Cohort Study.

### ART sales data

As of 31 May 2024, 99% (*n* = 9265) of individuals enrolled in the SHCS were on ART. ART coverage was defined as the proportion of ART prescribed in the SHCS relative to ART sales. Median monthly ART coverage and total ART coverage reflect, respectively, monthly median and total prescriptions over the study period (June 2017–May 2024).

**Fig. 3:**
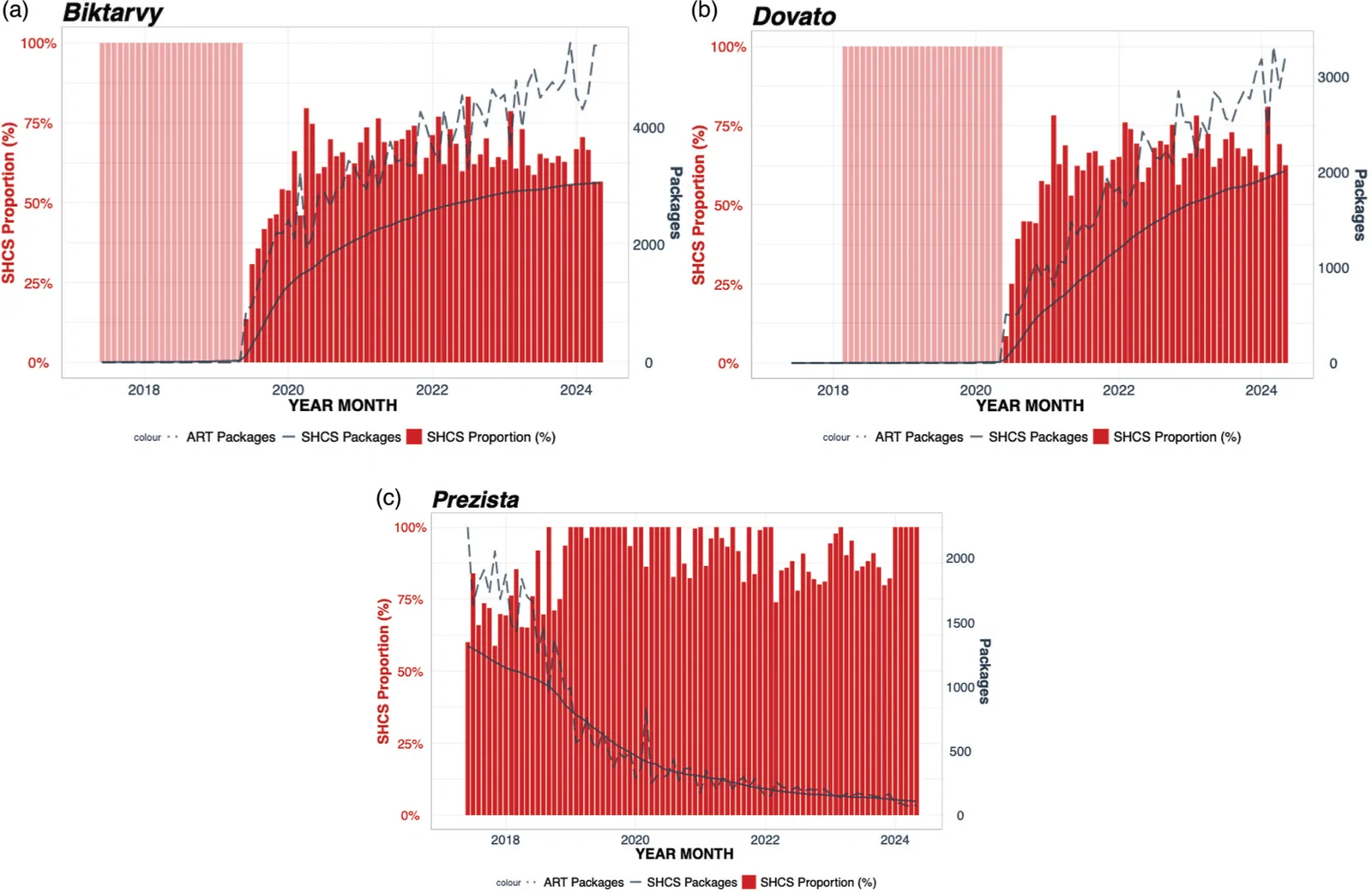
Monthly ART coverage for Biktarvy (BIC/TAF/FTC), Dovato (DTG/3TC) and Prezista (DRV), June 2017–May 2024.

Among the five most often prescribed single-tablet regimens, median monthly ART coverage ranged from 58% to 68%. Odefsey had the highest monthly median ART coverage (68%, IQR 63.4–77.2%), followed by Dovato (65%, IQR 60.5–69.6%), Biktarvy (64%, IQR 59.5–70.4%), Triumeq 64% (IQR 60.1–69.3%), and Genvoya (58%, IQR 54.1–64.8%; Fig. 3a, b, Figure S15, Table S1, Supplemental Digital Content). Total ART coverage was highest for Triumeq at almost 100% (Table S1, Supplemental Digital Content).

Monthly SHCS coverage (in %) is shown as bar plots on the left *y*-axis; monthly SHCS prescriptions (solid lines) and national ART sales (dotted lines) are shown on the right *y*-axis. The *x*-axis represents months; light shaded bars indicate periods when each ART was available through clinical trials before market authorization (Biktarvy: 12 April 2019; Dovato: 1 May 2020). A marked peak in Biktarvy and Prezista sales in early 2020 likely reflects private stockpiling during the COVID-19 pandemic. Abbreviations: SHCS, Swiss HIV Cohort Study; ART, antiretroviral therapy; BIC, bictegravir; DTG, dolutegravir; DRV, darunavir; FTC, emtricitabine; TAF, tenofovir alafenamide; 3TC, lamivudine.

Among salvage regimens, median monthly ART coverage ranged from 54% to 100%. Prezista had the highest median monthly ART coverage (100%, IQR 81.6–100%), followed by Retrovir (59%, IQR 46.2–75.0%), Intelence (57%, IQR 48.6–67.9%), and Celsentri (54% IQR 41.4–71.4%; Fig. 3c, Figure S16, Table S1, Supplemental Digital Content).

Overall, median and total ART coverage were similar across regimens, at about 64% for single-tablet regimens and 58% for salvage regimens.

### Targeted literature comparison

**Fig. 4:**
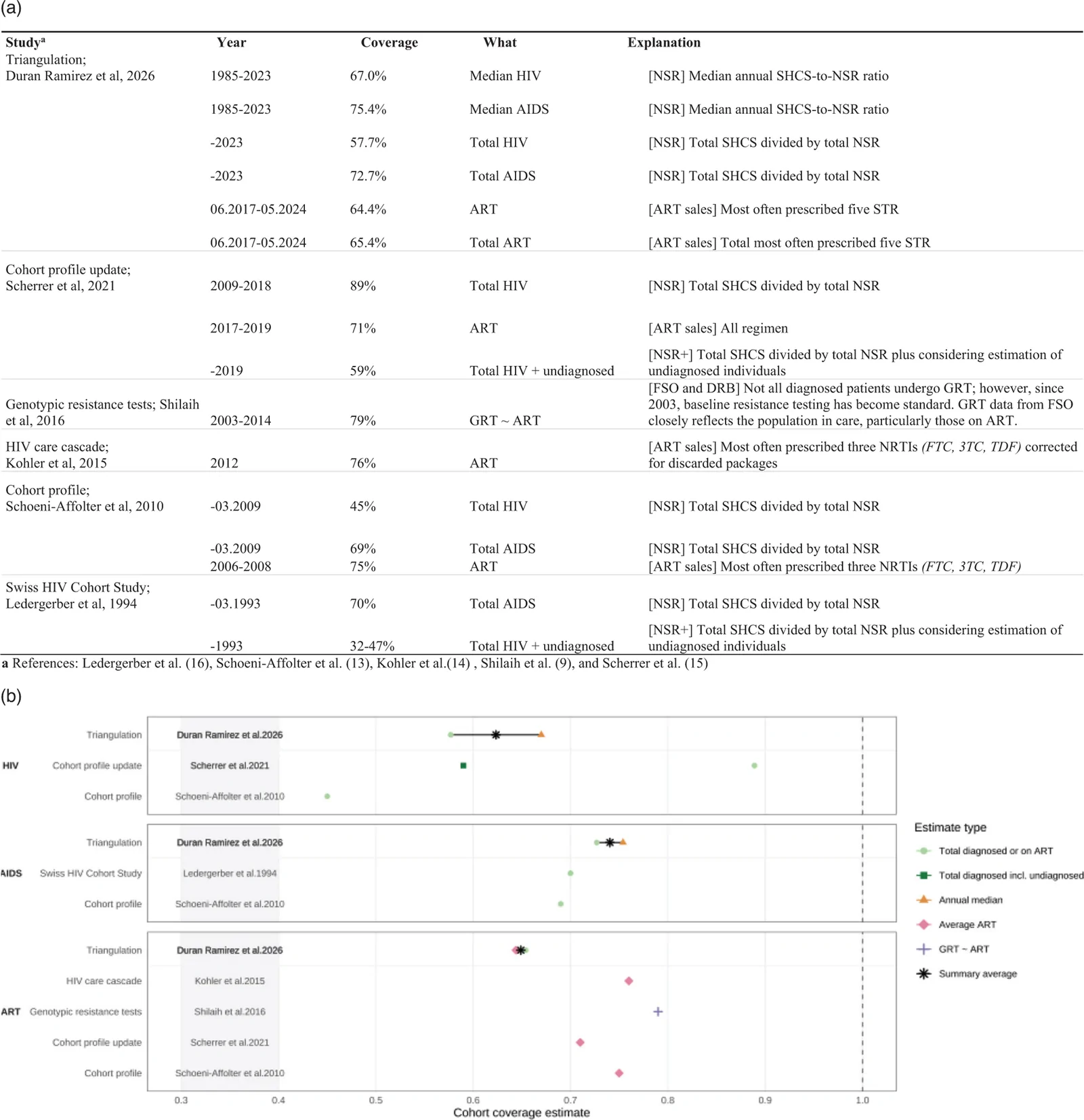
SHCS coverage estimates from data triangulation framework.

Five published studies have previously estimated coverage of the SHCS using diverse methodological approaches and national reference data sources (Fig. 4a, b). Reported estimates varied: HIV diagnoses coverage ranged from 45% to 89%, AIDS diagnoses coverage from 69% to 70%, and ART uptake coverage from 71% to 79%.

(a) Summary table with study characteristics and coverage explanations. (b) Coverage estimates with summary statistics, stratified by category. The *y*-axis shows different studies, targeted literature are shown below the solid line; *x*-axis represent cohort coverage estimates, with colour and shape indicating the estimation type. Abbreviations: AIDS, acquired immunodeficiency syndrome; ART, antiretroviral therapy; DRB, SHCS Drug Resistance Database, FSO, Federal Statistical Office, FTC, emtricitabine, GRT, genotypic resistance test; HIV; human immunodeficiency virus; 3TC, lamivudine; NRTI, nucleoside reverse transcriptase inhibitor; NSR, National Surveillance Report; SHCS, Swiss HIV Cohort Study; STR, single tablet regimen; TDF, tenofovir disoproxil fumarate.

The mean SHCS coverage was 62.4% for people diagnosed with HIV, 74.0% for people diagnosed with AIDS, and 64.9% for people on ART, corresponding to estimates derived from step 1 (HIV/AIDS diagnoses) and step 2 (ART uptake) of the data triangulation framework (Fig. 4b).

Previously published SHCS studies estimated higher total HIV diagnoses coverage for 2009–2018 (89% vs. 71.9%) and lower total HIV diagnoses coverage before 2009 (45% vs. 56.3%) compared with our estimates. Total AIDS coverage was similar (69% vs. 71.1% and 70% vs. 67.5%, Table S4, Supplemental Digital Content). These discrepancies likely reflect differences in definitions (e.g., enrolment vs. earliest diagnosis date), observation periods (e.g., partial vs. full years), and the use of updated data accounting for reporting delays. ART uptake coverage estimates were not compared because of differences in treatment regimens.

## Discussion

Over 38 years, the SHCS captured a substantial proportion of people diagnosed with HIV in Switzerland. Mean cohort coverage was 62.4% for HIV diagnoses, 74.0% for AIDS diagnoses, and 64.9% for ART uptake, based on estimates derived from the data triangulation framework. This degree of cohort coverage is remarkable for a voluntary research cohort, given that participation requires informed consent, enrolment through SHCS-affiliated providers, and is constrained by limited financial capacity to include all healthcare providers. Achieving this level of cohort coverage over nearly four decades reflects the strength of the SHCS infrastructure, its distributed clinical network, and its adaptability to changes in HIV care in Switzerland. By comparison, five European cohorts with varying inclusion profiles achieved 21.4–73.4% cohort coverage over a single decade [24].

Cohort coverage was slightly higher among individuals with AIDS-defining conditions, consistent with the SHCS's origins as a specialized tertiary-care network [16], where people with advanced disease were more likely to be referred and retained. This origin may also contribute to an overrepresentation of AIDS, as specialist providers are more experienced in diagnosing and reporting AIDS-defining conditions. Although enrolment has since broadened to include regional hospitals and private practitioners [13,15,16], future work should assess the extent of any remaining overrepresentation.

Our findings revealed a recent decline in SHCS-to-NSR ratios, likely reflecting several converging changes in HIV care and population dynamics. Advances in ART now allow many individuals with stable or recent diagnoses to remain in primary care, while treatment from private or non-hospital providers reduces enrolment in SHCS centres, contributing to lower cohort coverage, as reflected in some ART-based coverage estimates. Emerging psychosocial factors, including migration-related challenges (e.g., short stays), mental health issues, chemsex-associated behaviours [25], and cultural or language barriers, may further complicate engagement and retention. Although disengagement from care is relatively rare in Switzerland [15] and has declined over time, SHCS data show that over half of those interrupting for >14 months do not return, and many who re-engage present with advanced disease [26]. Consequently, future work should investigate whether the decline in cohort coverage primarily reflects changes in the care model and population structure or rather signals emerging gaps in cohort engagement.

Geographic heterogeneity was also evident, with lower cohort coverage in more decentralized regions such as Central and Midland Switzerland. This may partly reflect lower incidence in rural areas [18], but also points to structural factors such as limited institutional participation and reduced referral pathways. Improving equity will likely require regionally tailored strategies to engage private practitioners and smaller institutions.

The SHCS continued to capture the broad demographic profile of the diagnosed population [16]. Representativeness across key HIV acquisition modes (MSM, HET, PWIPS) has also remained remarkably stable, despite substantial changes in epidemic dynamics [13,15,16] clinical presentations, and treatment paradigms [27,28]. Such stability is uncommon among European HIV cohorts and reflects both the comprehensiveness of the SHCS and its integration within the Swiss health system [29]. However, differences remain. Females were slightly underrepresented by around 4% less than males, as in European cohorts [24]. PWIPS also tend to be underrepresented in some European cohorts (ranging from 2–5% less) [24], whereas the SHCS achieved comparatively better inclusion, possibly reflecting long-standing harm reduction policies [30] and close collaborations with dedicated services.

These differences highlight that cohort coverage and representativeness is not uniform across populations. Knowledge of cohort coverage and representativeness in subpopulations and time periods is therefore important when interpreting SHCS-based studies. These may vary depending on the study population and design, for example when including all cohort participants vs. only individuals on ART in recent years. For specific subgroups, such as females, the extent to which findings are generalizable to the overall diagnosed females with HET acquisition remains uncertain.

Looking ahead, the SHCS – like other established cohorts – faces the challenge of sustaining representativeness amid an ageing HIV population, migration, and evolving care models. Older adults and females are to some degree underrepresented, highlighting the need for future research into factors influencing cohort engagement. Monitoring cohort coverage and representativeness is therefore essential to identify gaps, track changes over time, and safeguard the cohort's continued role as a complement to national surveillance. From a public health perspective, the lack of national data on all diagnosed PWH limits the precision of cohort coverage estimation. While discussions on routine minimal data collection are ongoing, implementation has not yet been achieved. In the meantime, regular application of data triangulation, as demonstrated here, provides a practical interim solution.

Several limitations should be acknowledged. First, our analysis focused on people diagnosed with HIV, which is inherent to this type of analysis. Second, HIV/AIDS coverage may be affected by incomplete early reporting, as national surveillance was voluntary until the late 1980s. Third, demographic coverage and representativeness is constrained because external surveillance data are only available as single characteristics (e.g., sex, age, or acquisition mode), and not across categories. In addition, anonymization of patient data, together with SHCS informed consent and Swiss privacy regulations, prevents individual-level linkage of the data sources. As a result, more granular stratified analyses, such as for female PWID or migrant populations, are currently not possible. Fourth, for ART uptake coverage, the granularity of drug reporting in the SHCS, recorded per day rather than by treatment package, may lead to underestimation. In particular, drugs such as darunavir (DRV), which are sometimes used for post-exposure prophylaxis (PEP), could slightly underestimate DRV coverage due to higher sales; however, as PEP courses are short (up to one month), these instances are limited. Finally, targeted literature comparisons cover different time periods and contexts; observed differences may reflect variation in reporting periods, ART provision from different providers, and changes in the population over time, including ageing and potential disengagement from the SHCS, rather than true differences in cohort coverage.

In conclusion, using multiple independent data sources trough a data triangulation framework provides a practical approach for monitoring cohort coverage and representativeness. Such approaches help ensure that findings from cohort studies remain generalizable and continue to inform clinical care and public health responses in Switzerland and beyond.

## Acknowledgements

The authors thank the participants of the SHCS; the physicians and study nurses for excellent patient care; the data centre of the SHCS for data management; the coordination centre for administrative assistance. We thank the Swiss Federal Office of Public Health for discussions on HIV and AIDS surveillance data. We thank IQVIA AG and Gilead Sciences for providing access to IQVIA data. We also thank Gilead Sciences, ViiV Healthcare and Johnson & Johnson for granting permission to use their ART sales data.

Authors’ contribution: J.J.D.R., R.D.K., H.F.G., and K.K. contributed to the conceptualisation of the study. J.J.D.R. was responsible for data curation, data management, conducted the formal analysis, developed the software, performed the visualizations, and wrote the original draft of the manuscript, which was subsequently revised by K.K., R.D.K., and H.F.G. H.F.G. secured the funding for the project. The investigation, including patient enrolment and data acquisition, was carried out by I.A., M.S., G.W., A.Ca., M.Ca., L.E., P.S., A.R., H.F.G., A.F., A.Cu., P.B., B.H., H.K., C.F., P.E.T., C.B., P.K., M.D.R., M.H., F.T., J.T., R.F., and D.S. Methodological development was undertaken by J.J.D.R., H.F.G., and K.K. Project administration and coordination of the research activities were managed by J.J.D.R., H.F.G., and K.K. H.F.G. and K.K. provided both the necessary resources for conducting the study and supervision throughout the project. K.K. was responsible for data management at the Data Centre of the Swiss HIV Cohort Study. J.J.D.R. and K.K. have directly accessed and verified the underlying data reported in the manuscript. All authors contributed to the review and editing of the manuscript and approved the final version. All authors had the final responsibility for the decision to submit for publication.

Members of the Swiss HIV Cohort Study: Abela IA, Aebi-Popp K, Anagnostopoulos A, Bernasconi E, Braun DL, Bucher HC, Calmy A, Cavassini M (Chairman of the Clinical and Laboratory Committee), Ciuffi A, Dollenmaier G, Egger M, Elzi L, Fehr JS, Fellay J, Frigerio Malossa S, Fux CA, Günthard HF, Hachfeld A, Haerry DHU (deputy of “Positive Council”), Hasse B, Hirsch HH, Hoffmann M, Hösli I, Huber M, Jackson-Perry D (patient representatives), Kahlert CR, Kaufmann D, Keiser O, Klimkait T, Kouyos RD, Kovari H, Kusejko K (Head of Data Centre), Labhardt ND, Leuzinger K, Martinez de Tejada B, Marzolini C, Metzner KJ, Müller N, Nemeth J, Nicca D, Notter J, Paioni P (Chairman of the Mother & Child Substudy), Pantaleo G, Perreau M, Rauch A (President of the SHCS), Salazar-Vizcaya LP, Schmid P, Segeral O, Speck RF, Stöckle M, Surial B, Tarr PE, Trkola A, Wandeler G (Chairman of the Scientific Board), Weisser M, Yerly S.

Use of artificial intelligence (AI) tools: ChatGPT (OpenAI, version GPT-4.5) was selectively used to assist in improving the English grammar and clarity of the manuscript. No AI tools were used for data collection, data analysis, or for generating images or graphical elements. The authors retain full responsibility for the content, accuracy, and interpretation of the manuscript.

### Data availability

«The individual-level datasets generated or analysed during the current study do not fulfil the requirements for open data access: (1) The SHCS informed consent states that sharing data outside the studies is only permitted for specific studies on HIV infection and its complications, and to researchers who have signed an agreement detailing the use of the data and biological samples; and (2) the data is too dense and comprehensive to preserve patient privacy in persons with HIV. According to the Swiss law, data cannot be shared if data subjects have not agreed or data is too sensitive to share. Investigators with a request for selected data should send a proposal to the respective SHCS address (www.shcs.ch/contact). The provision of data will be considered by the Scientific Board of the SHCS and the study team and is subject to Swiss legal and ethical regulations, and is outlined in a material and data transfer agreement.»

In addition, the authors do not have permission to share datasets obtained from external sources.

Justification for the inclusion of more than ten contributors: We included contributors from across the SHCS network, including university centres, regional hospitals, institutions, and private practices, to ensure broad representation in data verification and interpretation. This broad representation was essential, given the study's reliance on long-term cohort data and centre-specific expertise. All authors contributed to the interpretation of the data, critically reviewed the manuscript, approved the final version, and agreed to take responsibility for the integrity and accuracy of the work.

Funding: This work was supported within the framework of the Swiss HIV Cohort Study by the Swiss National Science Foundation [grant numbers 201369 to H.F.G, and #33FI-0_229621 to A.R], and in addition by the Swiss National Science Foundation [grant number 218408 to K.K]. The data are gathered by the five Swiss University Hospitals, two Cantonal Hospitals, affiliated hospitals and private physicians (listed in www.shcs.ch//health-care-providers/).

### Conflicts of interest

J.J.D.R, outside this work, received research grants from Swiss HIV Cohort Study, Gilead Sciences and ViiV Healthcare for studies that J.J.D.R serves as principal investigator all paid to the institution. R.D.K. received grants from the Swiss National Science Foundation, the National Institutes of Health and Gilead Sciences all paid to the institution. I.A received grants from the Swiss HIV Cohort Study, the University of Zurich and Gilead Sciences all paid to the institution. M.B. received grants for his Institution by Gilead sciences and ViiV Healthcare.A.Ca. received research grants from Gilead Sciences and MSD for studies where she serves as local study site co-investigator (paid to the institution). M.Ca's institution received research grants from Gilead and expert opinion fees from Gilead, MSD and ViiV. MCa's institution received travel grants from Gilead and MSD. D.H. reports consultancies with AstraZeneca, Gilead, UCB and ViiV Healthcare; travel grant from Gilead; institutional funding from AstraZeneca, Gilead, GSK, A. Menarini Pharma, MSD, Pfizer and ViiV Healthcare. P.S.'s institution has received travel grants, congress and advisory fees from ViiV and Gilead unrelated to this work. D.S. has received travel grants, congress and advisory fees from ViiV and Gilead unrelated to this work. M.S. received grants for congress participation from MSD and Gilead, and payments for advisory board activities from MSD, Gilead and ViiV, all paid to his institution. P.E.T.'s institution reports unrestricted and educational grants from Gilead, ViiV, and MSD, and advisory fees from Gilead and ViiV, all outside the submitted work. G.W. received research grants from Roche Diagnostics and Gilead Sciences, as well as expert opinion fees from MSD, Gilead sciences and ViiV, all paid to his institution. A.R reports support to his institution for advisory boards and/or travel grants from MSD, Gilead Sciences, and ViiV, and investigator initiated trial (IIT) grants from Gilead Sciences. All remuneration went to his home institution and not to A.R. personally, and all remuneration was provided outside the submitted work. H.F.G. has received research grants from the Swiss National Science Foundation, Swiss HIV Cohort Study, Yvonne Jacob Foundation, University of Zurich's Clinical Research Priority Program, Zurich Primary HIV Infection, Systems.X National Institutes of Health, Gilead Sciences, ViiV Healthcare, Roche and the Gates foundation (subcontractor), paid to his institution; personal honoraria for data safety monitoring board or advisory board consultations from Merck, ViiV Healthcare and Gilead Sciences. K.K. received grants from the Swiss National Science Foundation paid to the institution. For the remaining authors none were declared.

## Supplementary Material

Supplemental Digital Content
