# Supplementary material for: Improving cohort coverage estimation using a data triangulation framework: the Swiss HIV Cohort Study example
Source: AIDS. 2026 Apr 10;40(9):1314–26. doi: 10.1097/QAD.0000000000004523 (PMC13566368; doi:10.1097/QAD.0000000000004523)
Supplement: Supplementary file 1 [file aids-40-1314-s001.docx]

**Supplement: “Improving cohort coverage estimation using a data triangulation framework: the Swiss HIV Cohort Study example”**

Duran Ramirez et al.

Table of Contents

[Methods	2](#_Toc225267090)

[National HIV and AIDS surveillance system	2](#_Toc225267091)

[Limitations of comparisons between SHCS and national surveillance report	6](#_Toc225267092)

[Workflow to harmonise Swiss ART sales with SHCS prescriptions	8](#_Toc225267093)

[Study population	9](#_Toc225267094)

[Figure S1. Flowchart to illustrate participants for the Steps 1 and 2.	9](#_Toc225267025)

[Demographical stratification	10](#_Toc225267095)

[Figure S2. Annual distribution of HIV (A) and AIDS (B) diagnoses by sex at birth.	10](#_Toc225267026)

[Figure S3. Seven major regions and constituent cantons of Switzerland.	11](#_Toc225267027)

[Figure S4. Annual distribution of HIV (A) and AIDS (B) diagnoses by age group.	12](#_Toc225267028)

[Figure S5. Annual distribution of HIV (A) and AIDS (B) diagnoses by HIV acquisition mode.	13](#_Toc225267029)

[Results step 1: National surveillance reports	14](#_Toc225267096)

[Demographics	14](#_Toc225267097)

[Figure S6. Annual proportions of (A) people diagnosed with HIV and of (B) people diagnosed with AIDS-defining conditions by sex of birth, 1985-2023.	14](#_Toc225267030)

[Figure S7. Annual representativeness of HIV (A,B) and AIDS (C,D) diagnoses by sex at birth, 1985–2023.	15](#_Toc225267031)

[Figure S8. Major region of residence HIV (A) and AIDS (B) coverage by year, 2014–2023.	16](#_Toc225267032)

[Figure S9.Annual proportions of (A) people diagnosed with HIV and of (B) people diagnosed with AIDS-defining conditions by age groups, 1985-2023.	17](#_Toc225267033)

[Figure S10. Annual proportions of (A) people diagnosed with HIV and of (B) people diagnosed with AIDS-defining conditions by HIV acquisition mode, 1985-2023.	18](#_Toc225267034)

[Figure S11. Annual distribution of HIV (A,B) and AIDS (C,D) diagnoses by age group and HIV acquisition mode.	19](#_Toc225267035)

[Table S2. Demographical representativeness for 1985–2023	20](#_Toc225267041)

[Sensitivity analyses	24](#_Toc225267098)

[Figure S12. Cumulative annual numbers (A,C) and proportions (B,D) of people with HIV (A,C) and of individuals diagnosed with AIDS-defining conditions (B,D), 1985-2023.	24](#_Toc225267036)

[Figure S13. Annual numbers (A) and proportions (B) of people diagnosed with HIV using a 2021 SHCS data extraction.	25](#_Toc225267037)

[Figure S14. Time between multiple AIDS defining conditions.	26](#_Toc225267038)

[Table S3: Demographic distribution of HIV/AIDS diagnoses from 2000-2023 and 2010-2023.^a^	27](#_Toc225267042)

[Results step 2: Pharmaceutical ART sales	29](#_Toc225267099)

[Figure S15. Monthly ART coverage for most often prescribed single-tablet regimens Odefsey (RPV/FTC/TAF), Triumeq (ABC/DTG/3TC) and Genvoya (EVG/c/FTC/TAF), June 2017-May 2024.	29](#_Toc225267039)

[Figure S16. Monthly ART coverage for salvage regimens Intelence (ETV), Celcentri (MRV), and Retrovir (ZDV), June 2017-May 2024.	30](#_Toc225267040)

[Table S1. Median and total ART coverage of regimens, Jun 2017-May 2024	31](#_Toc225267043)

[Results step 3: Targeted literature comparison	32](#_Toc225267100)

[Table S4. Recalculated total HIV-diagnosed and AIDS coverage by targeted literature time frames	32](#_Toc225267044)

[References	33](#_Toc225267101)

# Methods

## National HIV and AIDS surveillance system

In Switzerland, HIV and AIDS are notifiable diseases under national infectious disease legislation. The surveillance system is coordinated by the Federal Office of Public Health (FOPH) and relies on mandatory dual reporting by physicians and laboratories, each using separate notification forms.

Physicians are required to submit a clinical notification form, which includes demographic information, clinical details, presumed mode of HIV acquisition, and any AIDS-defining conditions. Laboratories must complete a separate laboratory notification form, which captures diagnostic information, including the test date, test type, results, and the identity of the ordering physician.

HIV infection and AIDS each have their own notification forms (can be found in the next 3 pages). If both conditions are diagnosed simultaneously, both forms must be completed. Notifications can be submitted electronically or via standardised paper forms. Data are pseudonymised but include a unique identifier to allow deduplication.

The FOPH uses these data for national epidemiological surveillance and public health research. The surveillance system records new HIV diagnoses, new AIDS diagnoses, and simultaneous HIV and AIDS diagnoses. Only the first AIDS-defining condition is reportable per individual, following the 1993 European AIDS surveillance case definition(1).

## Limitations of comparisons between SHCS and national surveillance report

Although both the Swiss HIV Cohort Study (SHCS, www.shcs.ch/clinicians-study-nurses-and-data-managers/forms/shcs-code-book/) and the national surveillance report (NSR) are valuable data sources for understanding the Swiss HIV epidemic, they differ substantially in their scope, structure, and reporting practices, which complicates direct comparisons. The SHCS includes only enrolled individuals who provided informed consent and focuses on people receiving longitudinal HIV care. In contrast, the NSR captures all notified HIV and AIDS diagnoses in Switzerland (www.bag.admin.ch/de/meldepflichtige-infektionskrankheiten-woechentliche-fallzahlen), regardless of care engagement or long-term follow-up. The NSR comprises both total counts and counts stratified by sex, canton of residence, age group, and mode of HIV acquisition.

Most SHCS data were collected prospectively; however, retrospective data were incorporated for a subset of participants initially enrolled in a post-marketing surveillance study prior to 1988(2,3). In contrast, NSR notifications are, by default, collected retrospectively. This fundamental difference in data collection may affect the timing and completeness of diagnoses, particularly in the early years of the HIV epidemic.

Due to privacy laws and anonymisation protocols, individuals cannot be linked between the SHCS and NSR. This prevents individual-level comparison of clinical or demographic characteristics. Differences in case definitions, data completeness, and classification of HIV acquisition modes may introduce systematic discrepancies. For example, the SHCS uses self-reported or physician-confirmed HIV acquisition modes. In the NSR, individuals reporting multiple HIV acquisition modes [e.g. Men who have sex with men (MSM) and people who inject psychoactive substance (PWIPS)] are classified according to hierarchical rules, typically as MSM. This practice may lead to overrepresentation of certain categories in surveillance data compared with the cohort.

Furthermore, the term “new HIV diagnoses” in the NSR does not always reflect new infections or even first-ever diagnoses. It refers to the first report of a person with HIV in Switzerland and may include individuals diagnosed abroad who later migrate and enter care. This limits comparability of HIV diagnosis trends and between datasets. For these individuals, clinicians do not automatically receive a notification form, unlike for new laboratory-confirmed diagnoses. No comparable mechanism exists for AIDS diagnoses. As a result, such cases may be underreported in the NSR but still captured in the SHCS. This may bias cohort coverage estimates and further complicate comparisons of HIV and AIDS trends over time.

Taken together, these differences must be carefully considered when interpreting HIV and AIDS coverage estimates and trends derived from combined data sources.

## Workflow to harmonise Swiss ART sales with SHCS prescriptions

(1) Brand names recorded in the SHCS were mapped to their corresponding product IDs and anatomical therapeutic chemical (ATC) codes. Only prescriptions within the study period (June 2017 to May 2024) were included.
(2) The total number of prescribed pills was calculated by multiplying the number of days on treatment by the daily dosing frequency and amount per administration.
(3) Monthly numbers of required packages were derived by dividing the total number of prescribed pills by the number of pills per package. These estimates were compared with monthly Swiss ART sales data.
(4) Median monthly and total ART coverage were calculated for each regimen. If the market authorisation date occurred during the study period, calculations of median monthly ART coverage began from that date to account for clinical trial periods.

## Study population

**Figure S1. Flowchart to illustrate participants for the Steps 1 and 2.**

Abbreviations: ART, Antiretroviral therapy

## Demographical stratification

Figure S2. Annual distribution of HIV (A) and AIDS (B) diagnoses by sex at birth.

(A, B) Percentage of diagnoses each year, stratified by sex at birth and data source (NSR vs SHCS). The x-axis shows calendar years; the y-axis shows the proportion of diagnoses per year; coloured by sex at birth. Note: Sex at birth was missing or unknown for 7.2% (NSR) and 0.03% (SHCS) of HIV diagnoses, and for 0.013% (NSR) and 0.049% (SHCS) of AIDS diagnoses. These missing values reflect exclusions applied in the analysis (w/o unknown). Abbreviations: NSR, National Surveillance Report; SHCS, Swiss HIV Cohort Study; w/o, without

Figure S3. Seven major regions and constituent cantons of Switzerland.

The map displays the seven major geographic regions of Switzerland as defined in this study, with corresponding cantons: 1 Lake Geneva Region (GE – Geneva, VD – Vaud, VS – Valais), 2 Midland (BE – Bern, FR – Fribourg, JU – Jura, NE – Neuchâtel, SO – Solothurn), 3 Northwestern Switzerland ( (AG – Aargau, BL – Basel-Landschaft, BS – Basel-Stadt), 4 Zurich (ZH – Zurich), 5 Eastern Switzerland (AI – Appenzell Innerrhoden, AR – Appenzell Ausserrhoden, GL – Glarus, GR – Grisons, SG – St. Gallen, SH – Schaffhausen, TG – Thurgau), 6 Central Switzerland) (LU – Lucerne, NW – Nidwalden, OW – Obwalden, SZ – Schwyz, UR – Uri, ZG – Zug), and 7 Ticino (TI – Ticino).

Figure S4. Annual distribution of HIV (A) and AIDS (B) diagnoses by age group.

(A, B) Percentage of diagnoses each year, stratified by age group and by data source (NSR vs SHCS). The x-axis shows calendar years; the y-axis shows the proportion of diagnoses per year; coloured by age group. Note: Age at HIV diagnosis was missing or unknown for 53.5% (NSR) of HIV diagnoses. These missing values reflect exclusions applied in the analysis (w/o unknown). Abbreviations: SHCS, Swiss HIV Cohort Study; NSR, National Surveillance Report; w/o, without

Figure S5. Annual distribution of HIV (A) and AIDS (B) diagnoses by HIV acquisition mode.

(A, B) Percentage of diagnoses each year, stratified by HIV acquisition mode and by data source (NSR vs SHCS). The x-axis shows calendar years; the y-axis shows the proportion of diagnoses per year; coloured by HIV acquisition mode. Note Mode of HIV acquisition was missing, unknown, or other for 56.7% (NSR) and 3.4% (SHCS) of HIV diagnoses, and for 3.9% (NSR) and 3.3% (SHCS) of AIDS diagnoses. These missing values reflect exclusions applied in the analysis (w/o unknown). Abbreviations: HET, Heterosexual contact; MSM, Men who have sex with men; NSR, National Surveillance Report; PWIPS, people who inject psychoactive substance; SHCS, Swiss HIV Cohort Study; w/o, without

# Results step 1: National surveillance reports

## Demographics

Figure S6. Annual proportions of (A) people diagnosed with HIV and of (B) people diagnosed with AIDS-defining conditions by sex of birth, 1985-2023.

SHCS data (purple for HIV, brown for AIDS) are compared to NSR data (grey) by sex of birth. Panels A and B show the annual SHCS-to-NSR ratios, representing annual SHCS coverage. The x-axis shows year of diagnosis or report. Abbreviations: SHCS, Swiss HIV Cohort Study; NSR, National Surveillance Report

Figure S7. Annual representativeness of HIV (A,B) and AIDS (C,D) diagnoses by sex at birth, 1985–2023.

Annual ratio of SHCS to NSR sex-specific distributions. The x-axis shows years; y-axis shows the ratio of SHCS to NSR distribution by sex at birth. A horizontal reference line at y = 1 marks equal representation; values above or below indicate over- or underrepresentation in SHCS. (B,D) Background shading marks HIV treatment eras: Pre-Treatment (1985–1987), Pre-HAART (1987–1995), HAART (1995–2008), Treat-All (2008–2019), and ART era (2019–2023). Abbreviations: SHCS, Swiss HIV Cohort Study; NSR, National Surveillance Report

Figure S8. Major region of residence HIV (A) and AIDS (B) coverage by year, 2014–2023.

Major regions are color-coded based on SHCS coverage. Abbreviations: SHCS, Swiss HIV Cohort Study; NA, no data

Figure S9.Annual proportions of (A) people diagnosed with HIV and of (B) people diagnosed with AIDS-defining conditions by age groups, 1985-2023.

SHCS data (purple for HIV, brown for AIDS) are compared to NSR data (grey) by age groups. Panels A and B show the annual SHCS-to-NSR ratios, representing annual SHCS coverage. The x-axis shows year of diagnosis or report. Abbreviations: SHCS, Swiss HIV Cohort Study; NSR, National Surveillance Report

Figure S10. Annual proportions of (A) people diagnosed with HIV and of (B) people diagnosed with AIDS-defining conditions by HIV acquisition mode, 1985-2023.

SHCS data (purple for HIV, brown for AIDS) are compared to NSR data (grey) by acquisition mode. Panels A and B show the annual SHCS-to-NSR ratios, representing annual SHCS coverage. The x-axis shows year of diagnosis or report. Abbreviations: HET, Heterosexual contact; MSM, Men who have sex with men; NSR, National Surveillance Report; PWIPS, people who inject psychoactive substance; SHCS, Swiss HIV Cohort Study

Figure S11. Annual distribution of HIV (A,B) and AIDS (C,D) diagnoses by age group and HIV acquisition mode.

Annual ratio of SHCS to NSR distributions by (A,C) age group or (B,D) HIV acquisition mode. The x-axis shows years; y-axis shows the ratio of SHCS to NSR distribution by age group or HIV acquisition mode. Coloured by age groups or HIV acquisition modes. A horizontal reference line at y = 1 marks equal representation; values above or below indicate over- or underrepresentation in SHCS. Abbreviations: HET, Heterosexual contact; MSM, Men who have sex with men; NSR, National Surveillance Report; PWIPS, people who inject psychoactive substance; SHCS, Swiss HIV Cohort Study

**Table S2. Demographical representativeness for 1985–2023**

| Variable |  | N SHCS | N NSR | SHCS % | NSR % | Ratio SHCS%/ NSR %**^a^** |
| --- | --- | --- | --- | --- | --- | --- |
| **HIV 1985–2023** |  |  |  |  |  |  |
| Sex at birth | Female | 5,879 | 10,867 | 26.59 | 28.38 | 0.94 |
|  | Male | 16,225 | 24,663 | 73.38 | 64.40 | 1.14 |
| Acquisition mode | MSM | 10,412 | 5,976 | 48.76 | 36.07 | 1.35 |
|  | HET | 7,241 | 7,474 | 33.91 | 45.11 | 0.75 |
|  | PWIPS | 3,346 | 2,782 | 15.67 | 16.79 | 0.93 |
|  | Perinatal | 143 | 185 | 0.67 | 1.12 | 0.60 |
|  | Haemophilia | 53 | 0 | 0.25 | 0.00 |  |
|  | Transfusion | 159 | 153 | 0.74 | 0.92 | 0.81 |
| Age groups (years) | 20 - 29 | 8,770 | 5,079 | 41.20 | 29.24 | 1.41 |
|  | 30 - 39 | 7,050 | 6,381 | 33.12 | 36.73 | 0.90 |
|  | 40 - 49 | 3,376 | 3,391 | 15.86 | 19.52 | 0.81 |
|  | 50 - 59 | 1,475 | 1,696 | 6.93 | 9.76 | 0.71 |
|  | 60 - 69 | 528 | 666 | 2.48 | 3.83 | 0.65 |
|  | 70+ | 87 | 158 | 0.41 | 0.91 | 0.45 |
| **AIDS 1985–2023** |  |  |  |  |  |  |
| Sex at birth | Female | 1,839 | 2,607 | 24.68 | 25.44 | 0.97 |
|  | Male | 5,611 | 7,635 | 75.31 | 74.51 | 1.01 |
| Acquisition mode | MSM | 3,429 | 3,520 | 47.59 | 35.76 | 1.33 |
|  | HET | 2,206 | 2,862 | 30.61 | 29.08 | 1.05 |
|  | PWIPS | 1,446 | 3,247 | 20.07 | 32.99 | 0.61 |
|  | Perinatal | 39 | 110 | 0.54 | 1.12 | 0.48 |
|  | Haemophilia | 23 | 0 | 0.32 | 0.00 |  |
|  | Transfusion | 63 | 104 | 0.87 | 1.06 | 0.83 |
| Age groups (years) | 20 - 29 | 1,481 | 1,922 | 20.05 | 19.03 | 1.05 |
|  | 30 - 39 | 3,100 | 4,249 | 41.98 | 42.07 | 1.00 |
|  | 40 - 49 | 1,660 | 2,270 | 22.48 | 22.48 | 1.00 |
|  | 50 - 59 | 769 | 1,074 | 10.41 | 10.63 | 0.98 |
|  | 60 - 69 | 302 | 438 | 4.09 | 4.34 | 0.94 |
|  | 70+ | 73 | 146 | 0.99 | 1.45 | 0.68 |

**a** > 1 overrepresentation, < 1 underrepresentation, ~1 similar representation

Abbreviations: HET, Heterosexual contact; MSM, Men who have sex with men; NSR, National Surveillance Report; PWIPS, people who inject psychoactive substance; SHCS, Swiss HIV Cohort Study

## Sensitivity analyses

Figure S12. Cumulative annual numbers (A,C) and proportions (B,D) of people with HIV (A,B) and of individuals diagnosed with AIDS-defining conditions (C,D), 1985-2023.

SHCS data (purple for HIV, brown for AIDS) are compared to NSR data (grey). Panels A and C show number of diagnoses. Panels B and D show the annual SHCS-to-NSR ratios, representing annual SHCS coverage. The x-axis shows year of diagnosis or report; Coloured by SHCS (purple or brown) and NSR (grey). The median cumulative annual coverage was 55.0% for HIV diagnoses (IQR: 51.6–57.8%) and 69.9% for AIDS diagnoses (IQR: 68.1–72.0%). Abbreviations: IQR, Inter Quantile Range; SHCS, Swiss HIV Cohort Study; NSR, National Surveillance Report

Figure S13. Annual numbers (A) and proportions (B) of people diagnosed with HIV using a 2021 SHCS data extraction.

SHCS data (purple for HIV) are compared to NSR data (grey). Panels A show number of diagnoses. Panels B show the annual SHCS-to-NSR ratios, representing annual SHCS coverage. The x-axis shows year of diagnosis or report; Coloured by SHCS (purple) and NSR (grey). Abbreviations: SHCS, Swiss HIV Cohort Study; NSR, National Surveillance Report

Figure S14. Time between multiple AIDS defining conditions.

Histogram showing the maximum time difference (in 6 months) between any two AIDS-defining conditions per individual (*n*=4203). Bars are grouped in 12-month intervals. The x-axis shows selected reference points in years for interpretability, y axis number of individuals.

**Table S3: Demographic distribution of HIV/AIDS diagnoses from 2000-2023 and 2010-2023.^a^**

|  |  | **HIV (2000+)** | | **HIV (2010+)** | | **AIDS (2000+)** | | **AIDS (2010+)** | |
| --- | --- | --- | --- | --- | --- | --- | --- | --- | --- |
| **Variable** | **Category** | **SHCS** | **NSR** | **SHCS** | **NSR** | **SHCS** | **NSR** | **SHCS** | **NSR** |
| Sex at birth | Female | 24.4% | 29.0% | 18.9% | 23.9% | 28.2% | 28.7% | 23.3% | 24.9% |
|  | Male | 75.6% | 69.8% | 81.0% | 74.9% | 71.8% | 71.3% | 76.6% | 74.8% |
| HIV acquisition mode | MSM | 53.6% | 41.1% | 61.7% | 50.1% | 40.6% | 31.9% | 47.1% | 39.5% |
|  | HET | 41.0% | 50.3% | 35.2% | 45.4% | 46.3% | 50.6% | 44.9% | 52.7% |
|  | PWIPS | 3.6% | 7.1% | 1.4% | 3.1% | 11.1% | 16.2% | 6.6% | 6.8% |
|  | Perinatal | 1.2% | 0.8% | 1.1% | 0.8% | 0.8% | 0.5% | 0.8% | 0.5% |
|  | Haemophilia | 0.0% | 0.0% | 0.0% | 0.0% | 0.2% | 0.0% | 0.2% | 0.0% |
|  | Transfusion | 0.7% | 0.7% | 0.6% | 0.5% | 1.0% | 0.9% | 0.4% | 0.5% |
| Age groups at diagnosis (years) | 20 - 29 | 28.1% | 22.7% | 26.9% | 20.2% | 11.2% | 10.0% | 9.4% | 8.5% |
|  | 30 - 39 | 36.3% | 35.8% | 32.4% | 32.0% | 32.2% | 30.8% | 23.2% | 21.6% |
|  | 40 - 49 | 21.3% | 23.4% | 22.2% | 24.5% | 30.0% | 31.3% | 29.4% | 30.4% |
|  | 50 - 59 | 10.0% | 12.2% | 13.2% | 16.1% | 16.6% | 17.4% | 24.2% | 25.2% |
|  | 60 - 69 | 3.8% | 4.8% | 4.5% | 5.7% | 7.7% | 7.8% | 10.4% | 10.5% |
|  | 70+ | 0.6% | 1.1% | 0.8% | 1.4% | 2.3% | 2.8% | 3.5% | 3.7% |
| Major regions of residence**^b^** | Lake Geneva Region | 27.2% | 28.6% | 26.6% | 28.6% | 25.8% | 16.7% | 23.0% | 19.3% |
|  | Midland | 14.0% | 17.0% | 13.4% | 16.8% | 13.5% | 12.6% | 14.4% | 15.3% |
|  | Northwestern Switzerland | 12.6% | 9.6% | 13.7% | 9.5% | 14.3% | 8.3% | 16.2% | 10.5% |
|  | Zurich | 30.0% | 24.9% | 29.5% | 26.6% | 28.3% | 17.5% | 29.0% | 22.2% |
|  | Eastern Switzerland | 8.0% | 7.2% | 8.6% | 7.7% | 10.4% | 7.2% | 10.2% | 7.9% |
|  | Central Switzerland | 3.7% | 5.1% | 3.1% | 5.3% | 3.8% | 4.3% | 3.1% | 5.5% |
|  | Ticino | 3.1% | 3.3% | 3.5% | 3.6% | 2.9% | 2.4% | 3.1% | 2.7% |
| **a** Proportional distribution per strata in %  **b** Definitions in Figure S2.  Abbreviations: MSM, men who have sex with men; HET, heterosexual contact; PWIPS, people who inject psychoactive substance; SHCS, Swiss HIV Cohort Study; NSR, National Surveillance Report | | | | | | | | | |

# Results step 2: Pharmaceutical ART sales

Figure S15. Monthly ART coverage for most often prescribed single-tablet regimens Odefsey (RPV/FTC/TAF), Triumeq (ABC/DTG/3TC) and Genvoya (EVG/c/FTC/TAF), June 2017-May 2024.

Monthly SHCS coverage (in %) is shown as bar plots on the left y-axis; monthly SHCS prescriptions (solid lines) and national ART sales (dotted lines) are shown on the right y-axis. The x-axis represents months; light shaded bars indicate periods when each ART was available through clinical trials prior to market authorisation (Odefsey: 22 May 2018). A marked peak in ART sales in early 2020 likely reflects private stockpiling during the COVID-19 pandemic. Abbreviations: 3TC, lamivudine; ABC, Abacavir; ART, Antiretroviral therapy; C, Cobicistat; DTG, Dolutegravir; EVG, Elvitegravir; FTC, Emtricitabine; RPV, Rilpivirine; SHCS, Swiss HIV Cohort Study; TAF, Tenofovir Alafenamide.

Figure S16. Monthly ART coverage for salvage regimens Intelence (ETV), Celcentri (MRV), and Retrovir (ZDV), June 2017-May 2024.

Monthly SHCS coverage (in %) is shown as bar plots on the left y-axis; monthly SHCS prescriptions (solid lines) and national ART sales (dotted lines) are shown on the right y-axis. The x-axis represents months. Abbreviations: ETV, Etravirine; MRV, Maraviroc; SHCS, Swiss HIV Cohort Study; ART, antiretroviral therapy; ZDV, Zidovudine.

**Table S1. Median and total ART coverage of regimens, Jun 2017-May 2024**

| **REGIMEN** |  |  | **SHCS^a^** | **MEDIAN COVERAGE^b^** | **TOTAL COVERAGE^c^** |
| --- | --- | --- | --- | --- | --- |
| BIC FTC TAF |  | Biktarvy | 28.4% | 64.4% (IQR: 59.5%–70.4%) | 64.6% (137,921 presc/213,552 sales) |
| DTG 3TC |  | Dovato | 19.1% | 65.3% (IQR: 60.5%–69.6%) | 65.4% (61,508 presc/94,052 sales) |
| RPV FTC TAF |  | Odefsey | 8.4% | 68.0% (IQR: 63.4%–77.2%) | 68.1% (68,504 presc/100,599 sales) |
| ABC DTG 3TC |  | Triumeq | 5.6% | 63.9% (IQR: 60.1%–69.3%) | 99.6% (109,083 presc/109,544 sales) |
| EVG/c FTC TAF |  | Genvoya | 4.9% | 58.4% (IQR: 54.1%–64.8%) | 59.8% (89,055 presc/148,927 sales) |
|  |  |  | **66.4% total** | **64.4% median overall** | **65.4% median total overall** |
| DRV |  | Prezista | 4.7% | 100% (IQR: 81.6%–100.0%) | 85.9% (42,288 presc/49,254 sales) |
| ETV |  | Intelence | 0.5% | 57.4% (IQR: 48.6%–67.9%) | 61.5% (13,366 presc/21,748 sales) |
| MVC |  | Celsentri | 0.2% | 54.2% (IQR: 41.4%–71.4%) | 52.5% (3,325 presc/6,328 sales) |
| ZDV |  | Retrovir | 0.1% | 59.3% (IQR: 46.2%–75.0%) | 39.0% (1,651 presc/4,231 sales) |
|  |  |  | **5.5% total** | **58.4% median overall** | **57.0% median total overall** |

**a** Percentages indicate the proportion of participants on each regimen relative to all ART records in the SHCS at the reference date, 1 January 2024 ( www.shcs.ch/about-shcs/shcs-key-data-tables/)

**b** Median monthly ART coverage: Median monthly prescribed packages relative to Swiss sales over study period( June 2017 to May 2024), adjusted for market authorisation dates

**c** Total ART coverage: cumulative prescribed packages relative to total Swiss sales during the study period (June 2017 to May 2024), adjusted for market authorisation dates

Abbreviations: ART, antiretroviral therapy; Presc, Swiss HIV Cohort Study prescription

# Results step 3: Targeted literature comparison

**Table S4. Recalculated total HIV-diagnosed and AIDS coverage by targeted literature time frames**

| **Study** |  | **Year** | **Coverage** | **What** |
| --- | --- | --- | --- | --- |
| Duran Ramirez et al. |  | 2009-2018 | 70.5% (66.1,77.3%) | Median HIV (IQR) |
|  |  | 2009-2018 | 71.9% (4030/5604) | Total HIV (SHCS/NSR) |
|  |  | -12.2009 | 56.3% (17 766/31 580) | Total HIV (SHCS/NSR) |
|  |  | -12.2009 | 71.1% (6493 /9126) | Total AIDS (SHCS/NSR) |
|  |  | -12.1993 | 67.5% (2916 /4320) | Total AIDS (SHCS/NSR) |
| Scherrer et al.**^a^** |  | 2009-2018 | 89% (4868/5476) | Total HIV (SHCS/NSR) |
| Schoeni-Affolter et al. **^a^** |  | -03.2009 | 45% (15624/XX) | Total HIV (SHCS/NSR) |
|  |  | -03.2009 | 69% (XX/XX) | Total AIDS (SHCS/NSR) |
| Ledergerber et al. **^a^** |  | -03.1993 | 70% (2124/3028) | Total AIDS (SHCS/NSR) |

**a** References: Scherrer et al. (4), Schoeni-Affolter et al. (5), and Ledergerber et al. (6)

Abbreviations: IQR, Inter Quantile Range, SHCS, Swiss HIV Cohort Study; NSR, National Surveillance Report, XX, unknown values, -, until

**References**

1.	Castro KG, Ward JW, Slutsker L, Buehler JW, Jaffe HW, Berkelman RL, et al. 1993 Revised Classification System for HIV Infection and Expanded Surveillance Case Definition for AIDS Among Adolescents and Adults. Clinical Infectious Diseases. 1993 Oct 1;17(4):802–10. doi:10.1093/CLINIDS/17.4.802 PubMed PMID: 1361652.

2.	[Anti-retroviral therapy of HIV-infection. With preliminary results of the Swiss postmarketing surveillance of zidovudine] - PubMed [Internet]. [cited 2025 Jul 21]. Available from: https://pubmed.ncbi.nlm.nih.gov/1904164/

3.	History and background SHCS – Swiss HIV Cohort Study (SHCS) [Internet]. [cited 2025 Jul 21]. Available from: https://www.shcs.ch/about-shcs/organisation-shcs/history-and-background-shcs/

4.	Scherrer AU, Traytel A, Braun DL, Calmy A, Battegay M, Cavassini M, et al. Cohort Profile Update: The Swiss HIV Cohort Study (SHCS). Int J Epidemiol. 2021 Aug 7;2021:1–12. doi:10.1093/IJE/DYAB141 PubMed PMID: 34363666.

5.	Schoeni-Affolter F, Ledergerber B, Rickenbach M, Rudin C, Günthard HF, Telenti A, et al. Cohort profile: The Swiss HIV cohort study. Int J Epidemiol. 2010 Oct 1;39(5):1179–89. doi:10.1093/ije/dyp321 PubMed PMID: 19948780.

6.	Ledergerber B, von Overbeck J, Egger M, Lüthy R. The Swiss HIV Cohort Study: Rationale, organization and selected baseline characteristics. Sozial- und Präventivmedizin SPM. 1994;39(6):387–94. doi:10.1007/BF01299670/METRICS PubMed PMID: 7817627.
